# Pro-arrhythmic Effect of the Vein of Marshall Ethanol Ablation: A Case Report of Perimitral Flutter After Vein of Marshall Ethanol Ablation

**DOI:** 10.19102/icrm.2023.14122

**Published:** 2023-12-15

**Authors:** Natee Sirinvaravong, Anthony W. Salmeron, Emile G. Daoud, Mahmoud Houmsse

**Affiliations:** 1 Wexner Medical Center, Ohio State University, Columbus, OH, USA; 2 Johnson & Johnson MedTech: Biosense Webster Inc., Irvine, CA, USA

**Keywords:** Coronary sinus ablation, ethanol ablation, perimitral flutter, pro-arrhythmic, vein of Marshall

## Abstract

The ligament of Marshall is an embryological remnant of the left superior vena cava that contains neural tissues shown to be an arrhythmogenic source of atrial fibrillation (AF). Vein of Marshall (VOM) ethanol ablation is an ablation technique that can potentially treat AF by targeting the ligament of Marshall. We report a case of a patient who developed a pro-arrhythmic effect related to VOM ethanol ablation, which manifested as a perimitral flutter.

## Case presentation

**Figure 1:**
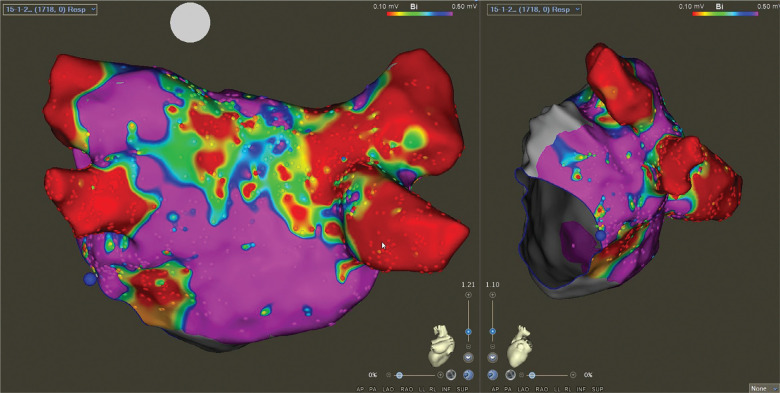
Baseline electroanatomic voltage map of the left atrium in the posteroanterior view (left panel) and the left lateral view (right panel).

**Figure 2:**
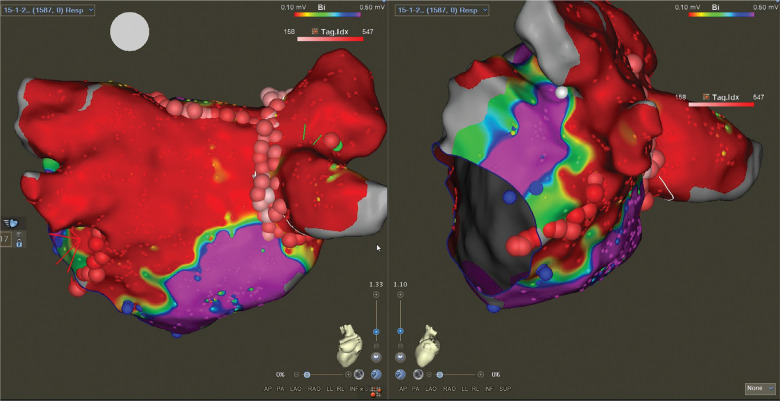
There is significant loss of voltage in the posterior wall, mitral isthmus, and left atrial coumadin ridge area after vein of Marshall ethanol ablation. The posterior wall along the ostium of the left pulmonary veins is isolated with no recordable atrial signals. Radiofrequency ablation was performed to complete right pulmonary vein isolation and linear roof and endocardial mitral isthmus ablation.

**Figure 3:**
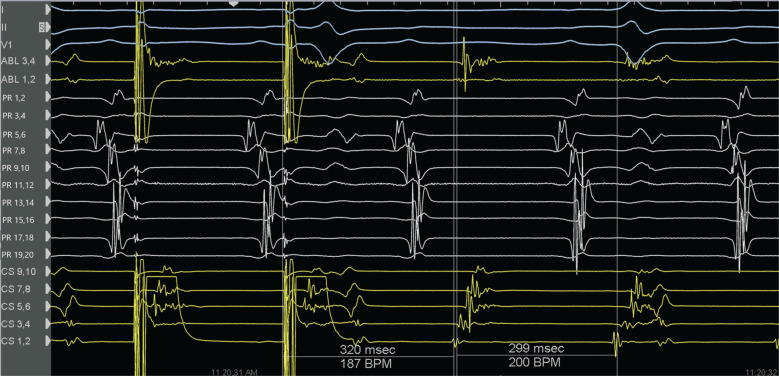
During atrial flutter, entrainment from the distal coronary sinus (CS) (electrode pair CS 1,2) showed concealed fusion with a post-pacing interval (PPI) of 320 ms, which is <30 ms longer than the tachycardia cycle length (299 ms). An excellent PPI was also obtained from the proximal CS (not shown in this figure) consistent with the perimitral atrial flutter. Electrocardiogram leads I, III, and V1; electrograms of distal ablation (ABL 1,2) and proximal ablation (ABL 3,4); electrograms of the PentaRay diagnostic catheter (Biosense Webster, Diamond Bar, CA, USA) (PR 1,2 through PR 19,20); and electrograms of the CS (proximal CS 9,10 through distal CS 1,2) are depicted. The PentaRay diagnostic catheter is positioned anterior to the left atrial coumadin ridge and proximal CS is at the CS ostium.

**Figure 4:**
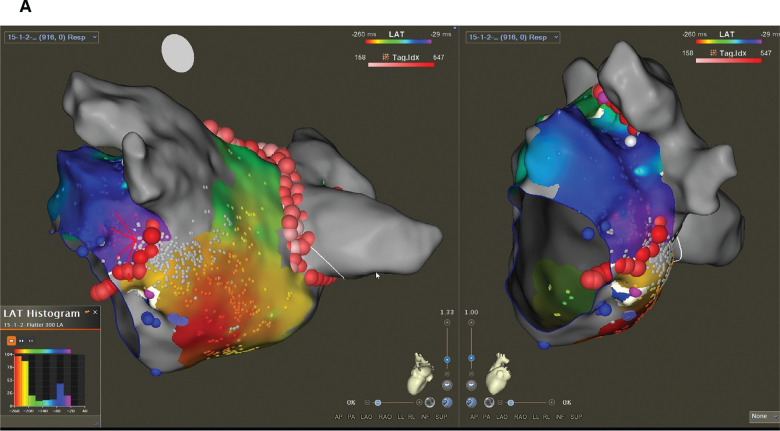

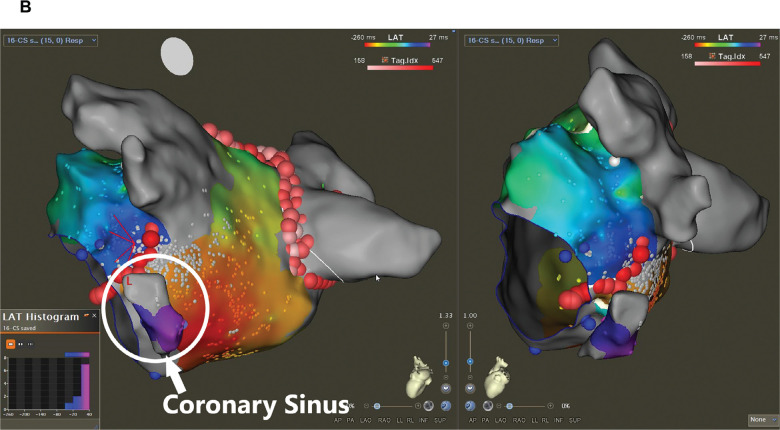
Activation mapping of the perimetral flutter cycle length (CL) (300 ms). **A:** The entire CL of the flutter recorded from the left atrial endocardium on the local activation time histogram is missing part of the CL from −20 to 40 ms. There is an endocardial conduction block across the mitral isthmus at the site of prior endocardial ablation. **B:** Additional activation mapping in the coronary sinus (white circle) identifies the missing part of the CL ([−20] to [40] ms on the local activation time histogram), which is the epicardial connection across the endocardial line of block.

**Figure 5:**
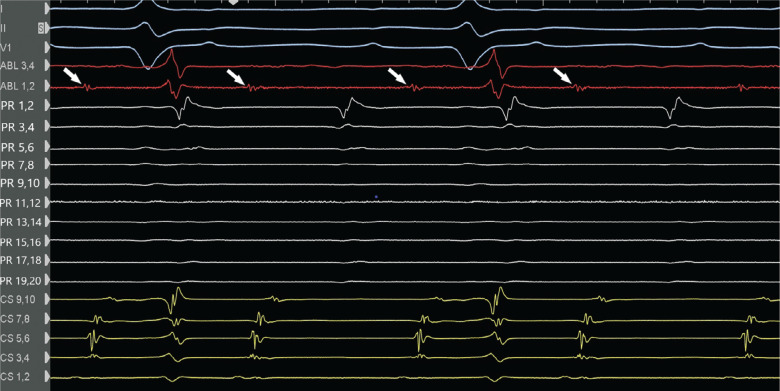
Mapping in the coronary sinus (CS) shows fractionated signals in the distal CS (white arrows). Ablation in this area terminated the perimetral flutter. Electrocardiogram leads I, III, and V1; electrograms of distal ablation (ABL 1,2) and proximal ablation (ABL 3,4); electrograms of the PentaRay diagnostic catheter (PR 1,2 through PR 19,20); and electrograms of the CS (proximal CS 9,10 through distal CS 1,2) are depicted. The PentaRay diagnostic catheter is positioned anterior to the left atrial coumadin ridge. The proximal CS 9,10 is positioned at the CS ostium.

A 59-year-old man presented for the ablation of persistent atrial fibrillation (AF). The pre-ablation voltage map in sinus rhythm is presented in **Figure 1**. Prior to left atrial ablation, the vein of Marshall (VOM) was identified by contrast injection in the coronary sinus (CS). The VOM was cannulated with an occlusion balloon, and 10 mL of 99% ethanol was injected. Radiofrequency ablation (RFA) was then performed to achieve bilateral pulmonary vein isolation as well as a linear roof and mitral isthmus (MI) ablation. The left pulmonary veins were isolated without the need for RFA along the posterior aspect due to the effect of VOM ethanol ablation **(Figure 2)**. Spontaneously, prior to confirming bidirectional conduction block across the MI, the rhythm changed to an atrial flutter with a tachycardia cycle length (TCL) of 299 ms. Entrainment from the proximal and distal CS showed a postpacing interval <30 ms longer than the TCL with concealed fusion, suggesting a perimitral flutter (PMF) **(Figure 3)**. Extensive activation mapping in the left atrium was not able to map the entire flutter cycle length (CL), with apparent endocardial MI block **(Figure 4A)**, suggesting the presence of an epicardial connection. Activation mapping in the CS demonstrated the epicardial connection across the MI, completing the missing part of the TCL **(Figure 4B)**. Ablation at the fractionated signals in the distal CS terminated the PMF **(Figure 5)**. Additional RFA was delivered in the CS until the confirmation of bidirectional MI block. There was no inducible flutter at the conclusion of the procedure. At 6 months of follow-up, the patient has not had any atrial arrhythmias.

## Discussion

The ligament of Marshall, located epicardially across the MI from the left atrial coumadin ridge to the CS at the level of the valve of Vieussens, is an embryological remnant of the left superior vena cava. Prior studies report that the ligament of Marshall in humans contains the VOM, sympathetic nerves, and multiple atrial myocardial fibers connecting the ligament to the CS musculature, left atrial myocardium, and pulmonary venous musculature.^1^ The ligament of Marshall is a potential cause of AF due to the presence of sympathetic neural tissues and has led to the structure being a target of ablation by VOM ethanol injection.^2^

PMF utilizes the MI as a critical portion of the macro–re-entry circuit. Ablation of PMF is achieved by creating a line of bidirectional conduction block across the MI, often between the left inferior pulmonary vein and the mitral annulus. This has been difficult to achieve with endocardial ablation alone due to various reasons, one of which is that the epicardial connection through the ligament of Marshall and CS can function as a bypass across the MI, facilitating PMF. Sakamoto et al. showed in a series of 50 patients that, after endocardial MI ablation, more than half of the patients had a residual connection in the CS or the Marshall tract.^3^ Previous studies have shown that ablation within the CS was needed in 67%–76% of the patients to achieve MI block.^4^ Ablation in the CS can be challenging due to poor blood flow limiting energy delivery, CS perforation, CS stenosis, or circumflex artery injury. Alcohol ablation of the VOM is a technique to target the epicardial connections across the MI and has been shown to facilitate MI ablation.^5^ MI endocardial ablation together with VOM ethanol ablation has been shown to achieve MI block in 93% of patients.^6^

In this case, the patient developed PMF after VOM ethanol ablation and endocardial MI ablation. Despite extensive ablation of the left atrial tissue with the ethanol injection **(Figure 2)**, the mapping of the PMF demonstrates that the critical component of the flutter is the remnant of unablated, slowly conducting low-amplitude fractionated signals recorded from the CS. This implies that the ethanol ablation resulted in a pro-arrhythmic effect and RFA at this site successfully terminated the PMF. We propose that VOM ethanol ablation resulted in a pro-arrhythmic effect for this patient by creating the substrate for PMF. In order to initiate and maintain a macro–re-entry circuit, there has to be a critical slow-conducting tissue. VOM ablation can eliminate part of the epicardial MI connection but leave behind heterogeneous and slowly conducting tissue that serves as a critical isthmus for PMF. This case demonstrates the importance of recognizing the epicardial connection across the MI both through the CS muscle sleeve and the bundle of Marshall in order to prevent and treat PMF.

## Conclusion

This case demonstrates that, even after VOM ablation, electrophysiologists should recognize that epicardial connection across the MI through the CS and the bundle of Marshall may persist and further ablation in this area may be required to treat PMF. VOM ethanol ablation can be pro-arrhythmic by creating a slowly conducting substrate across the MI for PMF. It is important to confirm bidirectional MI block after VOM ablation. Epicardial mapping and ablation in the CS is warranted after VOM ablation if MI block is not achieved after endocardial ablation.
